# Lived experiences of diabetic outpatients attending clinics in rural areas of Limpopo province in South Africa

**DOI:** 10.4102/hsag.v28i0.1726

**Published:** 2023-06-23

**Authors:** Mabitsela H. Mphasha, Tebogo M. Mothiba, Linda Skaal

**Affiliations:** 1 Department of Public Health, Faculty of Healthcare Sciences, University of Limpopo, Polokwane, South Africa; 2 Faculty of Healthcare Sciences, University of Limpopo, Polokwane, South Africa; 3 Department of Public Health, Faculty of Healthcare Sciences, University of Limpopo, Polokwane, South Africa

**Keywords:** diabetes, patients, living, experiences, diagnosis

## Abstract

**Background:**

Prevalence of diabetes mellitus is increasing in South Africa (SA), with many people unknowingly living with undiagnosed diabetes. Living with a long-term illness like diabetes significantly impacts every aspect of one’s life. It is essential to understand the lived experience of patients to ensure better management and intervention.

**Aim:**

To explore the lived experiences of diabetic outpatients.

**Setting:**

Clinics of Senwabarwana, in Blouberg Local Municipality of the Capricorn District Municipality in Limpopo province of SA.

**Method:**

Qualitative phenomenological exploratory descriptive study design was adopted to collect data from 17 diabetic patients. Purposive sampling was utilised to choose respondents. Data were collected through one-to-one interviews using voice recorders and field notes for nonverbal cues. Data were analysed using the eight steps of Tesch’s inductive, descriptive and open coding technique.

**Results:**

Respondents detailed difficulty disclosing their diagnosis due to feelings of shame. They also experienced stress and an inability to perform duties they used to perform before diagnosis. Male respondents detailed their experiences of sexual problems and a fear of losing their wives to other men as a result.

**Conclusion:**

Patients living with diabetes are unable to perform some tasks that they were able to perform before diagnosis. This could be attributed to poor dietary choices and a lack of social support, leading to patients missing critical elements of diabetes care. Quality of life of patients who are unable to perform their daily tasks should be assessed, with appropriate interventions introduced to curb further deterioration. Male diabetes patients experience sexual dysfunction and a fear of losing their wives, which exacerbates their stress.

**Contribution:**

This study encourages the adoption of a family-centred approach, partnering with family members in the care of diabetic outpatients since most of the care takes place at home. Further studies are also recommended to design interventions which would address the experiences of patients for better outcomes.

## Background

The increasing global prevalence of diabetes compromises the general well-being and quality of life (QoL) of patients. The International Diabetes Federation (IDF) (2021) reports that South Africa (SA) has an estimated 4.2 million adults (ages 20–79) diagnosed with diabetes mellitus (DM). However, over 1.5 million people in SA have undiagnosed DM (IDF 2019). Diabetes poses a burden to the psychological health and social lives of patients, and it causes symptoms of inadequate metabolic control, chronic complications and ultimately lifelong disabilities (Mohammadi et al. 2016; Raghavendra, Viveki & Gadgade 2017). Various studies have reported poor glycaemic outcomes among patients (Campos 2012; Raghavendra, Viveki & Gadgade 2017), resulting in microvascular and macrovascular complications (Campos 2012) which worsen the QoL (Trikkalinou, Papazafiropoulou & Melidonis 2017).

It has been found that the experiences of patients living with diabetes are influenced by various factors within their environment (Klu et al. 2022). These factors may either improve patients’ QoL or deteriorate it. It is important to also assess social determinants of health that may impact diabetes outcomes. Literature from studies conducted in the Africa region shows that patients living with diabetes often experience physical, psychological and social changes after diagnosis (Kalra, Jena & Yeravdekar 2018; Klu et al. 2022). Similar findings were reported in a qualitative study conducted in Eswatini, where it was reported that patients living with type 2 DM experienced physiological, psychological, socio-economic and self-management challenges related to their disorder (Khumalo et al. 2021). Moreover, it was found that patients living with diabetes experience a higher prevalence of disability attributed to complications (Kalyani et al. 2010). A South African study pointed out that a lack of knowledge can cause anxieties and frustrations in patients regarding their state of health and quality of care (Murphy et al. 2015). These often deteriorate their health and QoL. It is therefore critical to monitor patients regularly, detect diabetes-related complications sooner and take measures for better outcomes.

According to Holmström and Söderberg (2021), living with a long-term illness such as diabetes significantly impacts every aspect of patients’ lives. Studies on gestational DM found that women with the illness experienced negative feelings including fear, loss of normality and personal control, uncertainty and scepticism (Ge, Wikby & Rask 2017; Parsons et al. 2014). A review study indicated that factors such as cultural roles and beliefs, social stigmas, social and professional support, and social roles and barriers to self-care influence the experiences of women with diabetes (Devsam, Bogossian & Peacock 2013). Studies involving young persons with type 1 DM recognised that the condition is challenging and often requires healthcare, community and family support, including individual adjustments to manage everyday life and school (Holmström et al. 2017; Holmström, Häggström & Söderberg 2018). A South African study reported that the diagnosis of diabetes among African women is accompanied by complications such as mental illness, depression and infectious diseases (Mendenhall & Norris 2015). Type 2 DM patients experiencing diabetes-related distress are likely to be noncompliant to treatment, either due to denial or forgetfulness. The consequences of noncompliance are poor glycaemic control and complications (Snoek et al. 2011). Various studies have indicated that psychological variables such as acceptance, beliefs about diabetes, attitudes towards medical treatment and QoL are influential in the development and degree of diabetes-related distress (Ehrmann et al. 2015; Hu et al. 2007). Research indicated that it is essential to understand the lived experience of patients to improve health practice and interventions for better management of the disease (Ge, Wikby & Rask 2017; Lindseth & Norberg 2004), which is what this study aims to achieve.

## Research methodology

### Research design

A qualitative approach and phenomenological exploratory descriptive study design were applied, in which diabetic outpatients in rural areas of Limpopo were interviewed one on one to gain insight on their lived experience.

### Study setting

This study was conducted in clinics of Senwabarwana, a region in the Blouberg Local Municipality of the Capricorn District Municipality in the Limpopo province of SA. Senwabarwana is a rural area with a population of 162 297 (Statistics South Africa 2011). Blouberg Municipality is demarcated into 22 wards with a total of 22 physical clinics. However, there are wards with more than two clinics, while other wards have no clinics. The study setting was chosen because researchers wanted to have access to patients living with DM.

### Sampling and sampling size

The target population of this study is outpatients living with diabetes and receiving treatment in clinics of Senwabarwana. A total of eight clinics were chosen, consisting of two clinics per cluster. These clinics were chosen because they had many patients receiving diabetes treatment, which made it easier to identify respondents who met inclusion criteria. Thereafter, respondents were sourced from sampled clinics through verbal face-to-face engagements with potential respondents on clinic days when patients collected their treatment for diabetes and other chronic conditions. In each clinic, two respondents of different genders (except Clinic D, where both respondents were male) were sampled. In Clinic A, three respondents were sampled. The respondents were chosen because they could relay their lived experiences. Seventeen (17) respondents were purposively sampled, and the sample was reliant upon data saturation being reached. Data saturation was reached at participant number 15, who repeated what others had already indicated. Two additional patients were sampled but added no new information. On that basis, data collection was discontinued at respondent number 17. The inclusion criteria in the study incorporated patients with either type 1 or type 2 diabetes, who had lived with diabetes for at least 6 months, and who were over the age of 18 years.

### Data collection instrument and procedure

The data were collected through one-on-one interviews using voice recorders, with field notes to document nonverbal cues. Respondents were alerted when voice recorders were switched on and off. The interviews were done in Sepedi, which is a dominant language in the area. The grand tour question for the interview was ‘Kindly describe your experiences of living with diabetes post-diagnosis’. The data was gathered through an unstructured interview guide with open-ended questions which were developed based on available literature. A pilot study was conducted in nonparticipating clinics within Senwabarwana before the main study, with the purpose of pretesting the interview guide. The pilot study’s findings did not inform any modification on data collection instruments and were not included in the main study. The data from all respondents was collected by one researcher from eight clinics within Senwabarwana. Based on the responses to each question, further probing and clarity-seeking questions were asked to obtain more information. The researcher used statements such as ‘May you please tell more?’ or ‘Please let us talk more about that’ to encourage participants to give more information about their lived experiences. Interviews with respondents took 20–40 min, and the data collection process was completed within 2 months.

The researcher used bracketing, intuiting and reflective remarks during the interviews. For bracketing, the researcher laid aside what is known about lived experiences of respondents in avoidance of preconceived ideas and beliefs. For intuiting, the researcher adhered to the questions in the interview guide and remained naïve for the avoidance of his own views. Lastly, for reflection, the researcher reflected on interesting remarks from respondents to encourage them to elaborate, by stating that ‘So what you are actually saying is…’

### Measures to ensure trustworthiness

Measure of rigour in this study was ensured through trustworthiness by observing credibility, transferability, confirmability and dependability, as shown in Table 1 (Ramathuba & Ndou 2020).

**TABLE 1:** Measures of trustworthiness.

Strategy	Criteria	Applicability
Credibility	Prolonged engagement	The researcher interviewed respondents for about 20–40 min and probing was done to obtain in-depth information. The first author worked as a dietitian at the only hospital in Blouberg, which provided service delivery through outreach service to all clinics.
	Member checks	It was done at the conclusion of each interview, whereby the researcher summarised information and the respondent validated the accuracy.
Transferability	Data saturation	Data was collected until saturation was reached. Data saturation was reached with respondent 17.
	Thick description	A detailed description of the study setting, participants and methodology was provided and shall permit other researchers to transfer findings.
Confirmability	Peer review	The researcher independently coded and recoded data and also developed themes. The services of an independent coder were acquired to further analyse data, which ultimately led to a consensus meeting of the researcher and independent coder for confirmation of findings.
	Audit trail	The researcher explained to the independent coder how data was collected and analysed, and consensus was reached. All the materials, records and notes collected during the study will be kept safely for the next 5 years.
Dependability	Dense description of research methods	A full description of research methods and reporting of verbatim results makes the findings dependable.
	Reliance on data collection tools, independent coder and supervisors	The researcher relied on the voice recorder and field notes, as well as supervisors and an independent coder.

*Source:* Ramathuba, D.U. & Ndou, H., 2020, ‘Ethical conflicts experienced by intensive care unit health professionals in a regional hospital, Limpopo province, South Africa’, *Health SA Gesondheid* 25(0), 1–9. https://doi.org/10.4102/hsag.v25i0.1183

### Data analysis

The recorded semistructured interviews, conducted in Sepedi, were transcribed verbatim, translated to English and further presented to a language interpreter. The researcher began analysing transcripts and field notes after each data collection session. After the data collection and analysis by the researcher, the data transcripts and field notes were submitted to an independent coder. The researcher and independent coder met in a consensus meeting and conceded to themes and subthemes. Respondents’ direct quotations were made and caught in italic format to support findings. Data was analysed using the eight steps of Tesch’s inductive, descriptive open coding technique (Creswell 2014), as outlined below:

#### Step 1 – Reading through the data

The researcher made sense of the data by reading all the verbatim transcripts carefully and repeatedly. This gave them ideas about the data segments and what they meant. The researcher understood transcripts and jotted down ideas.

#### Step 2 – Reduction of the collected data

The researcher scaled down the data collected to codes based on the existence or frequency of concepts used in the verbatim transcriptions. The researcher then listed all topics that emerged during the scaling down and grouped together the similar topics, and those that did not have association were clustered separately.

#### Step 3 – Asking questions about the meaning of the collected data

The researchers asked self-questions about transcriptions of the interview, based on the codes which existed from the frequency of the concepts. Questions included ‘What is this about?’ and ‘What is the underlying meaning?’ The researcher was able to get the meaning of the data, which led to coding.

#### Step 4 – Abbreviation of topics to codes

The researcher abbreviated topics, which emerged as codes. These codes were written next to the appropriate segments of the transcription. All these codes were written in the margins of the paper.

#### Step 5 – Development of themes and subthemes

The researcher developed themes and subthemes from coded data and the associated texts and reduced the total list by grouping topics that related to one another to create meaning of the themes and subthemes.

#### Step 6 – Compare the codes, topics and themes for duplication

The researchers reworked from the beginning to check the work for duplication. There was no necessity to refine the codes, topics and themes.

#### Step 7 – Initial grouping of all themes and subthemes

The data belonging to each theme were assembled in one column and preliminary analysis was performed, which was followed by the meeting between the researcher, supervisors and co-coder to reach consensus on themes and subthemes that each one had come up with independently.

#### Step 8 – Recoding

There was no need for recoding.

### Ethical considerations

This study is part of bigger study approved by Turfloop Research and Ethics Committee (ref. no. REC-0310111-031). The permission to access patients in the clinics was given by Limpopo Department of Health (ref. no. LP 201903-007). All respondents voluntarily gave and signed written informed consent for participation in the study. The respondents were informed about their privileges to withdraw from the study at any stage without punishment and that their withdrawal would not affect diabetes treatment received from the facilities. None of the respondents withdrew from the study. Privacy of the respondents was maintained by interviewing patients in private consulting rooms of the clinics. Confidentiality was maintained by not capturing the real names of participants.

## Results

### Demographics of study respondents

Table 2 shows that 10 out of the 17 respondents were male. Eleven were 60 years of age and above, living with diabetes for 5 years or more. Lastly, from each facility two respondents of different genders (except in Clinic D) were sampled. In Clinic A, three respondents were sampled.

**TABLE 2:** Demographic profile of respondents.

Respondent number	Age	Gender	Years living with diabetes	Clinic
1	60	Male	Over 5 years	A
2	63	Female	Over 2 years	A
3	65	Male	Over 5 years	A
4	63	Male	Over 10 years	B
5	56	Female	4 years	B
6	64	Male	5–6 years	C
7	56	Female	8 months	C
8	58	Male	22 years	D
9	47	Male	2 years	D
10	91	Female	9 months	E
11	69	Male	23 years	E
12	54	Female	5 years	F
13	73	Male	5 years	F
14	52	Female	Over 3 years	G
15	80	Male	18 years	G
16	61	Female	14 years	H
17	84	Male	Since 1990s	H

### Themes and subthemes that emerged from the data

Table 3 shows that two themes emerged from the data. Theme 1 had five subthemes, while theme 2 only had two subthemes.

**TABLE 3:** Themes and subthemes that emerged from the data.

Themes	Subthemes
1 Experiences of patients living with diabetes	1.1 Denial and acceptance 1.2 Minimal or lack of sex drive and fear of losing their wives 1.3 Fear and stress 1.4 Ashamed of diagnosis and nondisclosure to extended family 1.5 Physical signs and symptoms of living with diabetes
2 Self-care practices and impact on daily life	2.1 Nondisclosure and ending up eating wrong food due to societal expectation 2.2 Inability to work like before

### Theme 1: Experiences of patients living with diabetes

Patients living with diabetes often experience complications that impact their self-management. A diabetes diagnosis is difficult to accept, which delays the start of treatment and subsequently affects or worsens patients’ QoL. Respondents indicated their experiences of living with diabetes as evident in the following subthemes:

#### Subtheme 1.1: Denial and acceptance

The respondents’ journey to accepting their diabetes diagnosis was not an easy one, as they reported that it included getting the views of others and consulting traditional healers and churches. Below are statements made regarding acceptance or denial:

Respondent 1:

‘At first, I didn’t understand when they told me I have diabetes. Even when nurses said so, I did not take them seriously until I accepted when I noticed that my manhood is no longer working, and I cannot have sex.’ (A1, 60 years old, male)

Respondent 12:

‘I had difficulty in accepting diabetes diagnosis, since I lived for 90 years without any chronic disease.’ (F1, 54 years old, female)

#### Subtheme 1.2: Minimal or lack of sex drive and fear of losing their wives

Sexual dysfunction is a serious complication of diabetes that is common among patients and disrupts their daily lives. Respondents reported sexual dysfunction having an impact on their mental health and also causing the fear of losing their wives. Below are some of the statements made:

Respondent 3:

‘I almost passed on as a result of erectile issue and powerlessness to engage in sexual activities with the lady I wedded. I was unable to accomplish some other work, due to the stress brought by erectile problems. However, I and my wife are done having children, we were doing sex for entertainment only. Notwithstanding, these days I’m somewhat better and able to sex her regardless of whether isn’t unreasonably good.’ (A3, 65 years old, male)

Respondent 4:

‘Diabetes took away my happiness. I at this point don’t engage in sexual activities. I’m married, yet I can go for a month without sex. I just do once a month, yet it is likewise poor. What stresses me is that my wife is more youthful, I’m apprehensive she may choose to have intercourse out of wedlock since she isn’t getting fulfilment from me. I’m additionally still youthful, as you can see me. I actually should engage in sexual activities. This thing of not engaging in sex, I’m apprehensive may kill me than diabetes can.’ (B1, 63 years old, male)

#### Subtheme 1.3: Fear and stress

Diabetes is an emotional and delicate condition, which may trigger associated complications and compromise mental health. This subtheme outlines views of participants regarding diabetes diagnosis causing fear, and stress, and is supported by the quotes below:

Respondent 2:

‘I refuse to eat fatty, sugary and salty food my son buy for me, for the fear of complication and death, because I still want to live longer for him.’ (A2, 63 years old, female)

Respondent 6:

‘I no longer attends other people’s events and ceremonies for the fear of complicating in front of people, since I have to wait for thirty minutes before I eat after injecting insulin. I now and then forget to eat and get reminded when I feel signs and side effects such as shakiness and dizziness and then eat, so when I’m home I can promptly eat when I experience these indications; however, at functions … I can immediately eat when I experience these symptoms, but at other people’s ceremonies, you will have to wait for lunch time.’ (C1, 64 years old, male)

Respondent 16:

‘I love eating sweet and salty food, but nowadays I try not to, though difficult. Life before diabetes diagnosis was nicer because I would eat anything without restriction, for example, chocolate, cool drinks, sweets, etc. I stay alone since the passing of my aunt, so I have to force to avoid these foods like for the fear of diabetes complications.’ (H1, 61 years old, female)

#### Subtheme 1.4: Ashamed of diagnosis and nondisclosure to extended family

Respondents indicated that their diagnosis is their secret, and they are comfortable disclosing to immediate family with whom they reside in the same household. Diabetes is often associated with complications such as sexual dysfunction, which is a source of shame for many men. Respondents in this study also pointed out that the reason for keeping their diagnosis secret is to avoid being shamed and stigmatised. Below are some of the statements the participants made:

Respondent 1:

‘I cannot go around telling people about my disease diagnosis; it is my secret and that of my family.’ (A1, 60 years old, male)

Respondent 3:

‘I also experience erectile dysfunction. Fortunately, my wife supported me through and through, remained loyal and never told people about my condition. Had she told people, I would have died, since that’s what I was largely worried about.’ (A3, 65 years old, male)

#### Subtheme 1.5: Physical signs and symptoms of living with diabetes

This subtheme outlines physical signs and symptoms experienced by diabetes patients. The following statements by the participants explain the experiences:

Respondent 1:

‘I’m experiencing sexual dysfunction, and as a result I’m unable to have sex with the woman I married. This gives me sleepless evenings; I am constantly stressed.’ (A1, 60 years old, male)

Respondent 6:

‘When I feel not well and unsteady, it implies I should grab something to eat since I’m using insulin to control diabetes, and after injecting, I should wait for about 30 minutes before I eat, of which I sometimes forget due to stress.’ (C1, 64 years old, male)

Respondent 7:

‘I have been living with diabetes since 1997. I would now be able to feel when diabetes isn’t controlled. I now and again eat cake and sweet things during festivities; when I experience shaking and pains, it will mean my sugar level is uncontrolled [*or*] high, then I will drink lot of warm water to chill it off. At the point when I rest, I put water in a flask.’ (C2, 56 years old, female)

Respondent 8:

‘I have been living with diabetes for long. This illness once in a while overwhelms medicine. At the point when it overwhelms medication, I get sick, dizzy and painful.’ (D1, 58 years old, male)

### Theme 2: Self-care practices and impact on daily life

Living with diabetes brings along well-being challenges, identified with upkeep of daily life, and self-care practices, which impact diabetes outcomes. This was apparent in the accompanying subthemes that emerged from the theme.

#### Subtheme 2.1: Nondisclosure and ending up eating wrong food due to societal expectation

Consumption of unhealthy meals or food threatens the QoL of patients, which is already compromised by the presence of diabetes. Diabetes patients ought to stick to dietary modifications. Nonetheless, respondents pointed out that they once in a while eat food which they aren’t supposed to due to societal and family expectations. Below are some of the claims the respondents made:

Respondent 1:

‘I don’t eat beef; however, I’m at times compelled to eat it at church occasions or others’ ceremonies, since I can’t go around telling people what I eat and what I don’t eat.’ (A1, 60 years old, male)

Respondent 3:

‘My wife constantly serves me chicken intestines, knowing very well that I’m not supposed to eat them, and when I won’t eat them and cook something different, we fight; in this way, for harmony at home, I simply eat whatever I’m given to eat.’ (A3, 65 years old, male)

#### Subtheme 2.2: Inability to work like before

The presence of diabetes impacts all aspects of life. Respondents in this study indicated their inability to work like prior diagnosis with diabetes. Below quotations of respondents are in support of the subtheme:

Respondent 2:

‘I’m no longer able to do heavy house chores but only manages to do light house chores such as cleaning the house, washing dishes and clothes.’ (A2, 63 years old, female)

Respondent 8:

‘I can’t do hard work on account of body weakness and dizziness, which I used to do before diagnosis; I used to work at the farms doing hard labour before diagnosis.’ (D1, 58 years old, male)

Respondent 12:

‘Preceding diagnosis with diabetes, I used to walk to my friend’s place in the village; be that as it may, these days I no longer able to. I just stroll inside my yard. I also can no longer sweep the floor for prolonged period.’ (F1, 54 years old, female)

## Discussion

The aim of this study was to explore the lived experiences of diabetic patients after their diagnosis. Respondents in this study reported that they find it difficult to accept their diabetes diagnosis. The impact of a diabetes diagnosis and its complications may resemble stages of mourning, which are denial, anger, bargaining, depression and acceptance (Stroebe, Schut & Boerner 2017). Similarly, a Brazilian qualitative study reported that the impact of diabetes diagnosis is met with a mixture of feelings, which includes preoccupation, panic and even anger, and consequently there is denial of the condition (Da Silva et al. 2018). The delay in acceptance of diagnosis could impact resumption of treatment and worsen the condition.

Respondents in this study reported sleepless nights and worries because of the inability to sexually satisfy their wives and fearing to lose their wives to other men. Respondents in this study were aged 60 years and above; therefore, this study affirms an Indian qualitative study that reported a decline in sexual desire, activity and function among women aged 60 years compared to men (Kalra, Subramanyam & Pinto 2011). A sub-Saharan African qualitative study highlighted that sexual dysfunction is a serious complication of diabetes and is linked with psychological issues (Da Silva et al. 2018). Ordinary sexual life is a significant part of relationships, and despite what is expected, the presence of sexual problems may affect the self-image, self-esteem (Cooper et al. 2018) and masculinity of men living with diabetes. The presence of sexual problems predisposes diabetic patients to a risk of many psychological conditions such as stress and depression (Kalra et al. 2018). The burden of stress, depression and other psychological conditions among patients living with diabetes increases globally with the increase of the prevalence of sexual problems (Cooper et al. 2018).

Respondents in our study reported that they experienced fear and/or stress resulting from their diabetes diagnoses. An Iranian experimental study reported similar findings regarding stress among diabetic patients (Zamani-Alavijeh et al. 2018). It is important to encourage diabetic patients to disclose their diagnosis to family members and encourage their participation or involvement in caring for them. Primary healthcare facilities should strengthen diabetes education and involve communities. It has been discovered that stress can be both a cause and consequence of diabetes (Brannon, Feist & Updegraff 2013). Diabetes is an emotionally demanding disease, which makes patients vulnerable to a variety of psychological conditions (De Groot et al. 2016). Psychological issues among diabetic patients are linked with concerns with getting proper treatment or communicating with healthcare providers (Wardian & Sun 2014). Some of the patients’ stress is related to coping with fluctuating blood glucose concentrations, and the continuous need to balance oral medications and/or insulin dosages with physical activity and food intake (Fisher et al. 2013). This could result in depression (Ghosh & Chatterjee 2013), poor eating habits (Al-Mountashiri et al. 2017) and fear of hypoglycaemia (lzahrani et al. 2019). A Saudi Arabian study indicated a prevalence of anxiety, stress and depression among diabetes patients (lzahrani et al. 2019). It has been reported that high stress levels are associated with a poor QoL (Zhang et al. 2019). There is a need to focus on the psychological well-being of patients by including family members who can provide diabetes self-management support. Family inclusion in diabetes care has been found to decrease psychological distress among patients (Rosland et al. 2010). It is also found to positively impact patients’ outcomes, improving their health and QoL (Gunggu, Thon & Lian 2016).

The findings of this study showed that postdiagnosis, patients experienced shame and a difficulty disclosing to extended family and community members because of the fear of people feeling sorry for them. Similar findings were reported in a study conducted in the Netherlands, that diabetic patients only chose to disclose their diagnosis to those they live with and seldom to more distant family members (Kohinor, Stronks & Haafkens 2011). It further pointed out that it is taboo among the Netherlands community to disclose the diagnosis publicly for fear of gossip, job discrimination and shame (Kohinor et al. 2011). The inability or unwillingness to disclose their diabetes diagnosis could be related to stigmatisation. Stigma has been identified as a barrier to diabetes self-care (Schabert et al. 2013). Respondents in this study reported that they experienced physical signs and symptoms such as shakiness, sleepless nights, dizziness and pains. These symptoms are linked with poor glycaemic control. Additional signs and symptoms associated with poor glucose control, which were reported by respondents in the study, include polyuria, polydipsia, polyphagia, weight loss, visual disturbances and/or ketosis (Bytomski & Moorman 2010). Most outpatient diabetes care happens in the home (Mphasha, Mothiba & Skaal 2021), so there is a need for primary healthcare providers to partner with family members in the care of patients to ensure better outcomes.

Respondents in this study reported that due to an established tradition of eating at other people’s ceremonies (parties, funerals, etc.), they consumed unhealthy meals because of nondisclosure. This shows that patients had knowledge on the importance of nutrition in diabetes management, which contrasts with studies reporting poor knowledge among diabetic patients (Breen et al. 2015; Sami et al. 2020; Veeri et al. 2019). Respondents reported knowledge of food not to eat and the consequences of eating unhealthy food, which affirms the usefulness and importance of nutrition, exercise and medication in diabetic treatment (Mphasha et al. 2021). There may be a need to assess other factors that hamper diabetic patients’ adherence to treatment and develop strategies that will minimise their impact. Respondents indicated an inability to perform work they used to perform before being diagnosed with diabetes, which implies that the daily lives of respondents were disrupted by the presence of diabetes. There is a need for intervention to improve the daily lives of patients and restore their ability to perform work. At the same time, this finding raises concerns on the QoL of the respondents. There is a need to conduct QoL assessment surveys. The World Health Organization (1998) regards QoL as an ‘estimation of well-being as well as the measurement of health and the effects of health care’.

## Conclusion

Patients with diabetes are in denial post-diagnosis, finding it difficult to accept the condition and subsequently delaying the start of treatment. After diagnosis, patients feel ashamed and develop fear and the stress of losing their wives to other men. Patients’ treatment plans are negatively affected by a reluctance to disclose their diabetes diagnosis, resulting in the consumption of unhealthy food due to societal traditions of eating at other people’s events and/or homes. Diabetic patients already experience physical signs associated with poor glycaemic control and an inability to perform work they could perform before diagnosis. This implies health deterioration or a poor QoL, so there is an urgent need for diabetic interventions to improve outcomes. This study encourages the adoption of a family-centred approach, partnering with family members in the care of outpatients since most of the care takes place at home. Further studies are also recommended to design interventions which would address the experiences of patients for better outcomes.

### Recommendations

This study recommends collaboration of primary healthcare providers with family members in the care of patients to improve diabetes outcomes and health of patients. However, a study to assess QoL of patients must be carried out in order to design appropriate intervention. Further research should focus on experiences of caring for diabetic patients, which incorporates perspectives of family members, communities and primary healthcare workers. Additionally, a study to assess the QoL of patients living with diabetes should be undertaken in order to design an appropriate intervention.

### Limitations

The findings of the study cannot be extended to wider populations of diabetic patients in Limpopo. However, they may be transferable to another context.
